# Supplementary material for: Evaluation of Anti-Thyroperoxidase (A-TPO) and Anti-Thyroglobulin (A-Tg) Antibodies in Women with Previous Hashimoto’s Thyroiditis during and after Pregnancy
Source: J Clin Med. 2024 Aug 2;13(15):4519. doi: 10.3390/jcm13154519 (PMC11313569; doi:10.3390/jcm13154519)
Supplement: Supplementary file 1 [file jcm-13-04519-s001.zip › jcm-3127397-supplementary.pdf]

Table S1 – Serum values of thyroid hormones and TSH evaluated at the first visit and after treatment of primary hypothyroidism - Pregestational

Case	total T3<br>ng/dL<br>**nc/no**	total T4<br>mg/dL<br>**nc/no**	FT4<br>ng/dL<br>**nc/no**	TSH<br>mIU/L<br>**nc/no**	**TRAb**<br>**U/L**<br>**nc**
1	107/167	5,6/7,4	0,7/1,2	15/2,1	inferior a 1,0 U/L
2	96/140	4,9/7,2	0,6/1,4	19/1,9	inferior a 1,0 U/L
3	71/143	4,3/8,3	0,5/1,3	21/1,8	inferior a 1,0 U/L
4	121/165	5,1/7,3	0,7/1,4	16/2,2	inferior a 1,0 U/L
5	101/172	6,8/9,1	0,8/1,3	21/1,9	inferior a 1,0 U/L
6	77/98	4,1/6,3	0,5/1,5	27/2,4	inferior a 1,0 U/L
7	79/165	5,6/9,2	0,6/1,3	21/1,9	inferior a 1,0 U/L
8	82/97	6,1/7,3	0,7/1,5	18/1,8	inferior a 1,0 U/L
9	75/105	4,9/8,3	0,4/1,5	29/2,1	inferior a 1,0 U/L
10	83/123	4,3/7,8	0,5/1,4	21/2,1	inferior a 1,0 U/L
11	74/98	4,1/6,9	0,5/1,3	25/1,9	inferior a 1,0 U/L
12	71/152	4,9/8,3	0,7/1,2	19/2.0	inferior a 1,0 U/L
13	68/162	4,1/9,3	0,3/1,4	35/1,8	inferior a 1,0 U/L
14	79/143	5,3/8,9	0,7/1,6	19/1,3	inferior a 1,0 U/L
15	71/101	4,9/8,3	0,6/1,4	18/1,2	inferior a 1,0 U/L
16	90/147	5,3/7,4	0,7/1,3	23/2,4	inferior a 1,0 U/L
17	102/176	6,2/8,3	0,5/1,1	27/1,9	inferior a 1,0 U/L
18	128/189	6,9/9,3	0,7/1,2	19/2,1	inferior a 1,0 U/L
19	74/165	4,9/7,1	0,5/1,4	28/2,1	inferior a 1,0 U/L
20	76/162	5,3/7,3	0,7/1,3	27/1,2	inferior a 1,0 U/L
21	65/142	3,9/8,3	0,3/1,2	39/1,9	inferior a 1,0 U/L
22	79/151	4,5/9/7	0,5/1,1	26/1,2	inferior a 1,0 U/L
23	80/149	4,2/8,5	0,6/1,5	25/0,9	inferior a 1,0 U/L
24	93/161	5,1/9/8	0,5/1,3	27/1,1	inferior a 1,0 U/L
25	76/143	4,7/7,4	0,7/1,1	19/1,7	inferior a 1,0 U/L
26	71/192	4,2/9/8	0,6/1,2	25/1,1	inferior a 1,0 U/L
27	69/169	4,6/83	0,3/1,4	41/2,4	inferior a 1,0 U/L
28	102/168	7,3/9,4	0,6/1,1	31/1,3	inferior a 1,0 U/L
29	74/167	7,4/9,1	0,7/,1,4	28/0,9	inferior a 1,0 U/L
30	75/145	6,9/8,9	0,6/1,3	27/1,0	inferior a 1,0 U/L

nc= first visit

no= after treatment

Reference values: total triiodothyronine (total T3):70 to 200 ng/dL; total thyroxine (total T4): 4.5 to 12.0 μg/dL; Free T4: 0.9 to 1.7 ng/dL; thyroid-stimulating hormone (TSH): 0.45 to 4.5 mIU/L; anti-TSH receptor antibody (anti-TSHR or TRAb); positive greater than 1.5 U/L and normal less than 1.0 U/L

Table S2- Serum values of thyroid hormones and TSH evaluated in the first trimester (n1), second trimester (n2), third trimester (n3) of gestation and after 3 months of the Postgestational period (n4) and TRAb at the first visit and after 3 months of the Postgestational period (n4).

Case	T4 livre<br>média ng/ dL<br>n1/n2/n3/n4	TSH<br>média mUI/ L<br>n1/n2/n3/n4	TRAb nc<br>média U/L<br>n4	T3<br>média ng/dL<br>total n4
1	1,1/1,0/1,2/1,3	0,9/1,2/1,5/2,1	inferior a 1,0	123
2	1,0/1,1/1,0/1,2	2,0/2,1/1,2/1,3	inferior a 1,0	94
3	1,3/1,1/1,0/1,2	1,4/1,8/2,1/1,9	inferior a 1,0	163
4	1,0/1,1/1,0/1,2	1,2/0,8/1,1/2,5	inferior a 1,0	143
5	1,3/1,2/1,0/1,5	0,9/1,9/2,1/2,9	inferior a 1,0	89
6	1,4/1,1/1,0/1,1	1,9/2,0/2,1/1,2	inferior a 1,0	104
7	1,1/1,3/1,2/1,4	1,2/1,9/0,9/2,8	inferior a 1,0	99
8	1,0/1,2/1,0/1,3	1,1/1,2/2,0/2,1	inferior a 1,0	178
9	1,3/1,1/1,0/1,6	1,0/0,9/2,2/2,9	inferior a 1,0	156
10	1,1/1,0/1,2/1,1	1,1/1,9/2,0/1,1	inferior a 1,0	143
11	1,2/1,1/1,0/1,6	1,0/1,1/2,1/2,9	inferior a 1,0	89
12	1,0/1,1/1,1/1,4	0,8/0,9/1,3/1,9	inferior a 1,0	91
13	1,4/1,1/1,1/1,5	1,1/1,9/2,0/2,9	inferior a 1,0	175
14	1,2/1,1/1,0/1,5	0,7/0,9/1,1/1,2	inferior a 1,0	102
15	1,1/1,0/1,2/1,6	1,1/1,8/2,6/2,1	inferior a 1,0	109
16	1,0/1,1/1,0/1,3	1,0/1,1/1,3/2,1	inferior a 1,0	92
17	1,3/1,1/1,0/1,2	0,9/0,8/1,2/1,1	inferior a 1,0	89
18	1,2/1,1/1,0/1,3	1,9/1,3/1,1/1,7	inferior a 1,0	172
19	1,4/1,2/1,0/1,4	1,0/1,1/1,2/2,0	inferior a 1,0	153
20	1,1/1,0/1,1/1,3	0,9/1,0/2,1/2,9	inferior a 1,0	149
21	1,0/1,1/1,1/1,6	1,0/1,1/2,1/2,2	inferior a 1,0	155
22	1,1/1,0/1,1/1,6	1,6/2,1/2,5/2,9	inferior a 1,0	90
23	1,0/1,0/1,1/1,2	1,1/0,9/1,2/2,2	inferior a 1,0	98
24	1,1/1,2/1,0/1,4	0,8/2,2/2,1/2,8	inferior a 1,0	86
25	1,0/1,0/1,1/1,3	1,2/1,1/1,9/2,0	inferior a 1,0	163
26	1,1/1,0/1,0/1,2	1,1/1,0/0,9/2,9	inferior a 1,0	162
27	1,0/1,1/1,1/1,4	1,0/0,9/2,1/2,9	inferior a 1,0	179
28	1,0/1,3/1,1/1,5	0,8/1,0/1,1/2,4	inferior a 1,0	97
29	1,1/1,1/1,0/1,2	1,0/2,1/2,5/2,1	inferior a 1,0	142
30	1,2/1,1/1,0/1,3	0,9/1,2/1,7/2,8	inferior a 1,0	155

nc= first visit

n1 - first trimester

n2 - second trimester

n3 - third trimester of gestation

n4 - Postgestational

Reference values: FT4 (free T4): n1: 0.9 to 1.5; n2: 0.7 to 1.3; n3: 0.6 to 1.2; n4: 0.9 to 1.7 ng/dL;

thyroid stimulating hormone (TSH): n1: 0.1-2.5; n2: 0.2-3.5; n3: 0.3-3.5; n4: 0.45 to 4.5 mIU/L; anti-TSH receptor antibody (anti-TSHR or TRAb): positive greater than 1.5 U/L and normal less than 1.0 U/L; total triiodothyronine (total T3): 70 to 200 ng/dL

Table S3- Serum values of antithyroid antibodies A-TPO and A-Tg evaluated Pregestational in the three trimesters of gestation and three months Postgestational

Cases	A-TPO - IU/mL					A-Tg - IU/mL				
	Pre gestational	First trimester (average)	Second trimester (average)	Third trimester (average)	3 months Post gestational	Pre gestational	First trimester (average)	Second trimester (average)	Third trimester (average)	3 months Post gestational
1	1000	916	282	40	819	600	420	20	20	50
2	645	43	30	20	540	343	15	12	19	414
3	935	452	32	23	1000	452	43	21	9	642
4	215	116	106	94	343	171	34	22	28	215
5	937	251	21	8	892	248	192	32	29	362
6	3000	312	102	43	1000	390	103	34	19	638
7	3029	164	121	125	1600	116	56	45	56	128
8	734	123	34	5	897	870	18	24	12	678
9	3000	234	19	12	1000	435	231	13	9	546
10	479	121	65	16	569	198	32	12	5	768
11	3000	234	34	25	980	345	132	23	19	569
12	868	231	127	57	1000	662	158	48	35	1000
13	540	522	90	66	980	1300	188	288	187	1000
14	446	101	26	26	408	236	200	20	20	455
15	448	137	72	18	740	223	42	40	39	540
16	1000	54	54	29	1000	2600	210	120	32	600
17	254	132	94	30	540	152	60	34	14	270
18	211	164	127	54	388	220	115	70	30	23
19	3000	98	45	29	768	345	121	89	23	456
20	1000	534	15	14	1000	458	408	61	49	540
21	600	254	94	54	1000	547	153	35	37	651
22	3000	236	121	34	910	456	209	34	5	789
23	3000	548	234	64	3000	3000	245	124	23	3000
24	678	474	123	41	548	457	92	47	32	687
25	389	298	105	51	254	178	197	87	34	154
26	3000	958	451	154	3000	3000	754	124	75	3000
27	897	571	297	78	578	574	124	35	24	989
28	390	301	293	10	450	505	412	71	21	674
29	1000	854	542	101	1000	1000	540	354	40	1000
30	3000	901	400	124	3000	3000	897	300	89	3000

Reference values: A-TPO – antithyroperoxidase antibody – negative when lower than 35 IU/mL; A-Tg - antithyroglobulin antibody - negative when lower than 40 IU/mL
